# ‘Learning to shape life’ – a qualitative study on the challenges posed by a diagnosis of diabetes mellitus type 2

**DOI:** 10.1186/s12939-019-0924-3

**Published:** 2019-01-24

**Authors:** Astrid Fink, Eva-Maria Fach, Sara Lena Schröder

**Affiliations:** Martin-Luther-University Halle-Wittenberg, Medical Faculty, Institute of Medical Sociology, Magdeburger Str. 8, 06112 Halle (Saale), Germany

**Keywords:** Diabetes mellitus type 2, Adapting life, Patient education, Life style, Self-care, Qualitative study

## Abstract

**Background:**

Diabetes mellitus type 2 is a central challenge for health policy and healthcare in all advanced countries. For the affected persons, living with a diagnosis of type 2 diabetes is difficult because the disease and its treatment have a considerable effect on daily life. The aim of this study was to investigate the challenges associated with a diagnosis of type 2 diabetes for those affected and the range, depth and complexities of the subjective perspectives of the patients under the conditions of the German healthcare system.

**Methods:**

A cross-sectional qualitative study was conducted using a sample of 19 adult patients with type 2 diabetes mellitus. Patients were recruited successively from two specialized diabetological practices, three general practitioner’s offices, and two hospitals. The patients were interviewed once in person using semi-structured interviews. All interviews were recorded, transcribed, and analysed based on grounded theory.

**Results:**

Persons affected by diabetes mellitus type 2 seem to feel responsible for managing their disease. Two strategies of action could be identified: 1) patients strictly followed the recommendations of the physicians, or 2) they showed that they are knowledgably managing their diabetes mellitus type 2. The action strategy to address the disease seemed to be influenced by patients’ confidence in themselves, the effectiveness of the interventions, or the patients’ locus of control. Minor differences in educational status could be discovered, and patients who were less educated tended to follow the recommendations of the physicians very strictly and seemed to place more emphasis on being compliant, which goes hand in hand with a life with prohibitions and restrictions. In contrast, being perceived as competent patients who make their own rules to manage the disease in daily life appeared to be more important for people with higher education levels.

**Conclusion:**

Patient education and self-management programmes for diabetes mellitus type 2 should take different types of learners into account. Giving less-educated patients specific recommendations for successful diabetes self-management is particularly important.

**Trial registration:**

German clinical trial register (DRKS-ID: DRKS00007847).

**Electronic supplementary material:**

The online version of this article (10.1186/s12939-019-0924-3) contains supplementary material, which is available to authorized users.

## Background

Diabetes mellitus type 2 (T2DM) is a central challenge for health policy and healthcare in Germany and other advanced countries [1, 2]. This fact is first attributable to the frequency of the disease: the Diabetes Atlas of the International Diabetes Federation (IDF) estimated that the global prevalence of diabetes mellitus among adults aged 20–79 years was approximately 8.8% (CI_95_ 7.2–11.3%) in 2017 with a rate of 6.8% in European countries and 11.0% in North American and Caribbean regions. Of these diabetes cases, 90% were classified as type 2 diabetes [3]. In Germany, the prevalence of T2DM could be estimated at 7 to 8.6% of adults on the basis of population-related surveys and billing data from individual health insurance funds. The results varied depending on the age group studied and the database used [2, 4]. Second, the special significance of the disease is also due to the increased mortality from the disease. The proportion of people who died from diabetes in 2017 before the age of 60 was 32.9% in European countries and 45% in Northern America or Caribbean regions. The age and sex standardized mortality rate ratio among persons aged 45 years or older was 1.82 (CI_95%_ 1.45 to 2.28) for T2DM in Germany [5]. In addition to the challenges posed to the healthcare system, living with a diagnosis of T2DM is difficult. T2DM is a chronic condition that has a considerable effect on daily life. On the one hand, affected persons should implement the lifestyle modifications and if necessary, drug treatment, that the physician has recommended to them. On the other hand, people who have been diagnosed with T2DM have to take personal responsibility for managing the illness, and they have to find a responsible way to live with it [6, 7]. To live with the disease and avoid complications, which can be life threatening, people with T2DM need to learn self-management techniques and complex care activities [6–9]. In addition to these challenges, it is sometimes aggravating that patients receive misinformation from friends, relatives, and the media/internet on how to best manage diabetes [10].

Cross-disease self-management can be understood as a patient’s ability to address the symptoms, treatment, physical and psychosocial consequences, and lifestyle changes associated with a chronic disease [11, 12]. Self-management is considered to be of central importance in the treatment of T2DM [13, 14]. Diabetes self-management encompasses all activities where patients are involved in caring for their disease, promoting health, expanding their physical, social and emotional resources and preventing the long- and short-term effects of diabetes [14]. Specifically, this management includes healthy eating and physical activity, sustainable weight loss, foot care, and knowledge of glycaemic control. Some of these techniques depend on the form of individual therapy and the body mass index of the affected person [6, 7, 15]. These tasks show that a diagnosis of T2DM suddenly confronts a patient’s life with additional challenges of routines and everyday actions. Patients’ perspectives on the self-management of T2DM were presented in a meta-syntheses by Gomersall et al. 2011 [13]. The authors concluded that the development of diabetes-related experiences are dependent on social and political conditions and that diabetes care should pay attention to the internal world of persons with T2DM.

Structured patient education and self-management programmes are available for learning self-management. National and international expert associations recommend making structured diabetes education accessible to all persons with T2DM [16–18]. The training courses are aiming at patient empowerment, i.e., those affected learn to actively influence the course of diabetes by acquiring knowledge about health-promoting behaviours such as healthy nutrition, exercise and cardiovascular risk factors. This knowledge should lead to the successful implementation of diabetes-specific therapy requirements in everyday life [6, 17, 19, 20]. In addition, people with T2DM gain knowledge about secondary illnesses as well as the ability to recognize signs of complications (e.g., self-examination of the feet for early detection of diabetic foot syndrome). Structured and quality-assured patient education training is an internationally recognized measure with proven positive effects on parameters relevant to therapy such as blood sugar, glycated haemoglobin A1c (HbA1c) [21–24], blood pressure and body weight [6, 9, 25], as well as indicators of good quality of care, e.g., the frequency of screening for diabetic retinopathy [22, 25]. Several systematic reviews examining the effectiveness of self-management programmes found robust data demonstrating the effectiveness of these programmes evidenced by a statistically significant decrease in HbA1c levels [26]; HbA1c, fasting blood glucose, body weight, waist circumference, triglyceride levels and diabetes knowledge [27]; and HbA1c, diabetes knowledge, and self-care practices with short-term to long-term follow-ups [28].

Evidence indicates that persons with low socioeconomic status (a composite measure of an individual’s economic and sociological standing in relation to others) and T2DM have more barriers to diabetes self-management that are less pronounced [29, 30] and that they participate less frequently in diabetes education programmes [31, 32]. Furthermore, low individual socioeconomic status or residential area deprivation is often associated with worse process indicators and worse intermediate outcomes [33, 34]. The prevalence of T2DM in population groups with low socioeconomic status is also higher [35–37], and thus, these groups are often doubly disadvantaged (higher prevalence of T2DM and worse course of disease). These socioeconomic differences in treatment and the demand for considering diabetes care within the context of every patient [13] lead to the conclusion that diabetes-related experiences should be considered to be dependent on social conditions as well as on the specific features of the healthcare system [13]. Diabetes care should, as already noted, pay attention to the “inner” world of persons affected by T2DM.

To the best of our knowledge, no qualitative study in Germany has yet investigated the challenges associated with the diagnosis of T2DM for those affected and the range, depth and complexities of the subjective perspectives. To address these issues, a qualitative health care study was conducted from the patients’ point of view. Through analysing the patients’ experiences with their chronic disease and with treatment, this study aimed first, to explore and understand the challenges associated with a diagnosis of T2DM and how people adjust to the diagnosis and incorporate it into their lives under the conditions of the German healthcare system and second, to assess whether there were differences in patient experiences according to the different educational backgrounds of the participants, as the state of research suggests.

## Material and methods

### Study design

This exploratory qualitative study was based on the elements of grounded theory [38]. This research was considered suitable for capturing the subjective experiences of persons with T2DM within their social and cultural contexts and reviewing these experiences to arrive at an understanding of individuals’ perceptions of the disease and their adjustment to the diagnosis. The study was conducted as a single-centre, cross-sectional qualitative study in Halle/Saale, Saxony-Anhalt, Germany, a high-risk area for T2DM morbidity [39].

### Research team

Interviews were conducted by three female members of the team (AB, AF, EMF), who were researchers at the Institute of Medical Sociology. AB is a young medical anthropologist, and AF and EMF are experienced medical sociologists (AF: PhD in rehabilitation medicine and EMF: MA in sociology). All of them had undergone training in qualitative methods. Prior to her scientific career, the principal investigator (AF) worked as a dietician in healthcare, and in this capacity, she was responsible for educating patients with T2DM about nutrition. MW, a male student assistant, supported the project throughout its course.

### Participant recruiting

Participants were recruited from ambulatory medical practices (three general practitioners, one of them in rural area and two urban specialists in diabetology) and two hospitals (one urban and one rural). The inclusion criteria for participating in the study were 1) being 18 years and over; 2) having a diagnosis of T2DM; and 3) being able to speak and understand German. Two sampling methods were combined to use the strengths of the respective methods [40]. In keeping with the principles of theoretical sampling, the participants were recruited successively during alternating phases of data collection, development of theoretical categories and additional data collection [38]. Depending on the state of category and theory development, a decision was made by the team about which person should be interviewed next. Socioeconomic inequalities in prevalence, secondary diseases and mortality of T2DM are known to negatively affect socially disadvantaged individuals [35, 41–45], and persons with low socioeconomic status participate less frequently in studies [46]. Therefore, a qualitative sampling plan was additionally used to ensure that men and women from different socioeconomic groups would be represented. We decided to use education as a proxy for socioeconomic status. Participants were assigned to a group with high or low education based on their years of formal education and occupational training qualification. The classification was based on German epidemiological standards for the measurement and quantification of sociodemographic characteristics in epidemiological studies [47]. During the study, health care professionals identified eligible participants and gave brief information about the study. The patients who agreed to be contacted by the study team received a telephone call from a researcher who answered their questions and scheduled the interviews. Nineteen adult patients with T2DM participated in the study between July 2015 and June 2016, at which point theoretical saturation was reached. Because the first screening was held in doctors’ offices or hospitals, no statements can be made about patients refusing to participate.

### Participants

Nineteen patients were interviewed: 7 women and 12 men. They ranged in age from 47 to 87 years, and all were of German nationality. Their characteristics are summarized in Table 1.

**Table 1:** Patients’ characteristics (*n* = 19)

Variable	Grouping	
Age	Mean	63 years
	Minimum	47 years
	Maximum	87 years
Sex	Female	7
	Male	12
Education	Low	10
	High	9
Occupational status	Working	10
	Out of work	2
	Pensioners	7
Recruited in	General practice	9
	Special diabetological office	7
	Inpatient care	3
Therapy	Oral antidiabetic medication	9
	Insulin therapy	9
	Mixed form	1

### Data collection

The interviews were conducted face-to-face in a private, quiet room in the hospital, an office at the university or the participant’s home. They lasted 25–80 min and were audiotaped with the interviewees’ permission. No repeat interviews were conducted. Prior to the interview, participants were given an information sheet and a verbal explanation of the study and were encouraged to ask any questions. Then, the participants provided written informed consent. Interviews were semi-structured around a topic guide and included narrative-generating questions on the history and treatment of diabetes and its impact on daily life.

The topic guide was tested with four persons with T2DM. The purpose of the pre-test was to examine whether narratives were created and to elicit the participants’ reactions to the questions. The topic guide was revised after the pre-test and throughout the study, following the iterative process of data collection and analysis of grounded theory. To gather the participants’ sociodemographic data, questions about age, sex, nationality, marital status, education, professional qualifications, and occupation were raised subsequent to the interview. A situation report was prepared for each interview, and first memos were recorded after the conversation.

### Data analysis

A transcription agency transcribed the recordings verbatim. Pseudonyms were used to protect personal data. We used MAXQDA software for data management and analyses. We followed grounded theory analysis [48]. Open coding of the data began with the first two authors. The data were broken down into discrete parts, examined and compared for similarities and differences, and then clarified in discussions of prominent themes that had emerged. The transcripts were analysed by being coded into categories, which were then reduced by pattern coding (grouping categories into conceptual sets). The axial coding procedure was structured by Strauss and Corbin’s coding paradigm. Aspects of this paradigm are the causal conditions that lead to the phenomenon, the attributes of the context of the phenomenon, additional intervening conditions, action strategies to handle the phenomenon and the consequences of actions. This procedure led to carefully elaborated categories from which the theory is formulated in the course of selective coding. All authors (AF, MW, and AMF) iteratively conducted open and axial coding, and the principal investigator (AF) conducted selective coding by using abductive reasoning. The development and final application were discussed by the research team and in additional interpretation workshops. The phenomena were derived from the data and not determined in advance. Internal quality and credibility were attained by having three researchers independently code the data and by obtaining consensus through analysis and in discussions at additional interpretation workshops. A professional translation agency translated the passages from the interviews cited in this paper.

### Ethics and patients’ consent

Full ethical approval for the study was granted by the Ethical Review Committee of the Medical Faculty at Martin-Luther-University, Halle-Wittenberg. Participation was voluntary, and participants were informed that they could withdraw from the study at any point without giving reasons. They were also informed that the data would be pseudonymized and that the results might be published in a scientific journal. All participants gave written informed consent prior to the interview.

## Results

**Fig. 1:**
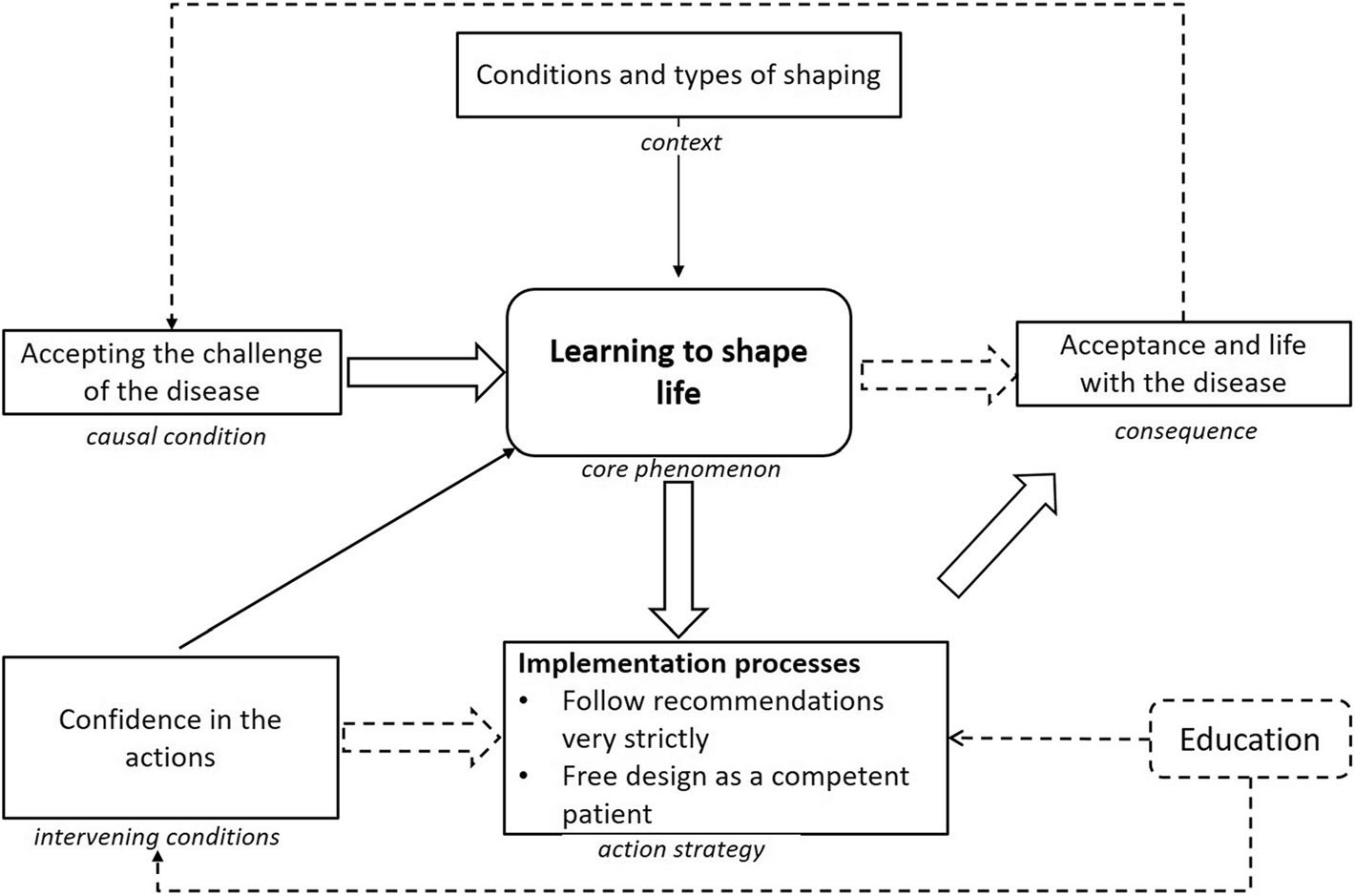
Categories and their connections of “learning to shape life”

Through exploring the patients’ experiences with their chronic disease, T2DM, and with treatment, we found that the focus was on how to cope with the disease in everyday life and how to adapt the life. In this result section, we first describe the path of “learning to shape life” (our developed model, Fig. 1) and then show small differences in adaptations in people with different education levels.

Diabetes mellitus type 2 affected both professional and private life. A central challenge for persons who received a diagnosis of T2DM seemed to be the management of the disease in daily living. In addition to adjustments in professional and private life, people needed to learn a great deal about self-care activities related to disease management. These included adapting one’s lifestyle in terms of physical activity and nutrition as well as the use of medication. The patient managed the adaptation well if he or she had sufficient knowledge about the disease, secondary diseases and measures to influence the disease. The professional health care system remained on the theoretical level in dealing with the disease, whereas the affected person was responsible for the implementation. This process of learning to shape life was the central phenomenon drawn from the narratives of the participants interviewed.

The main reason for “learning to shape life” seemed to be “accepting the challenge of the disease”. People with T2DM seemed to feel responsible for managing their disease. This feeling may have been influenced by the contextual aspects “conditions and types of shaping”**.** The action strategy of patients with T2DM could be influenced by the core phenomenon and by their own “confidence in the action” (intervening condition). This confidence seemed to be determined by the patients’ confidence in themselves, the effectiveness of interventions, or the patients’ locus of control. The two action strategies can be described as follows: 1) patients very strictly followed the recommendations of the physicians, or 2) they showed that they were knowledgably managing their T2DM. The consequence of the action strategies and learning to shape life could result in “acceptance and life with the disease”**.** As a chronic disease that is not always stable, T2DM requires adjustments. For this reason, the model of the path from the consequence “acceptance and life with the disease” is drawn back to the “accepting the challenge of the disease”. The categories and their connections are shown in Fig. 1, the derivation of the categories in Additional file 1.

### “Learning to shape life”

During the analysis, “learning to shape life” emerged from the interviews as the core phenomenon (story line). Learning how to shape one’s life is an ongoing process. With changes in illness or life situations, new situations arise to which one must react, so that the process may never be completed.

*Because it’s not just diabetes, it’s also going into the professional and private spheres, because such diabetes doesn’t, as they say so nicely, allow life to be so pronounced, but changes the body and everything. Yes, that’s not from eating habits, it goes all the way to, let’s say, even sexual. That’s all connected with it. (Participant 6, male, 50 years old).*

So and then I had a job with a security company, also a *security company, there I had to supervise alone night shifts 12 h things. And where they found out about diabetes and so on, they said, the doctor said it already, so: “For God’s sake, you’re not in a position, to do that”. […] I also have, as I said, great difficulties with the job centre there a suitable job again, because you want to get back into work, but there are great problems, because there are so great restrictions from the doctor. (Participant 15, male, 60 years old).

*Well, but that’s just one of those areas where I say, yes, on the one hand I know physical activities, food and so on. And the/The ball, I always have the feeling, the ball is with me, I would have to do something there, but really then just to play the ball again, there are a bit of difficulties. But I don’t think that’s necessarily a question for the practice or the doctors now, but that’s also the case, maybe also a little bit, I see it a little bit as a personal matter, where I say I’m on the radar. (Participant 3, male, 50 years old).*

### Accepting the challenge of the disease, conditions and types of shaping

The causal condition of learning to shape one’s life seemed to be accepting the challenge that the disease brings to the person who has received the diagnosis.

*“Yes, Dr. Martin put it simply then: ‘First we try with tablets, and if they don’t work and it gets worse, you’ll have to inject.’ That was a clear warning which made me pay attention.” (Participant 13, male, 72 years old)*

*“I take note of that and say: Well, then you have to stick to it. I felt that way then, your mother had it now, you have it now too. Well, that/yes, I have this/I did not panic or something and thought: You have to! I just took note of that.” (Participant 9, female, 80 years old)*

We found that there was a learning process to shaping one’s life to adjust to T2DM. This contextual factor raised questions about the conditions and types of such shaping. What motivations were there for learning? What encouraged learning? What hindered it? In the narratives of our participants, we found two different motivations for accepting the challenges of the disease. One of the motivations was linked to the negative emotion of fear, namely, “learning out of fear”. The other motivation was associated with an optimistic point of view, namely, “learning to keep going”. Fear seemed to be a motivator for learning and adaptation. Many of our participants had a family history of diabetes mellitus, and they reported that when they were diagnosed, they immediately thought something such as *diabetes put my mother in a wheelchair* (participant 6, 50 years old) and *I do not want that, so I have to do something* (participant 16, male, 47 years old). Fear affected several aspects in our model. First, it affected the causal condition “accepting the challenge of the disease”**.** Second, fear could be considered an intervening or maintaining condition. Finally, fear could be considered as a context because the patient’s knowledge of the disease affected his or her actions. Our participants expressed fears of an increase in therapy, secondary diseases, loss of independence, or loss of social ties.

*“And that is the problem that I get such problems with the legs by the sugar because the experience I had to make through my mother, who was also diabetic, for almost over 30 years and ended up in a wheelchair. I don’t want to go there.”* (Participant 6, male, 50 years old)

*“So, thank God, I do not need to inject. I also hope that I will not have to.” (Participant 1, female, 76 years old)*

The issue we describe as “learning to keep going” focused on having as few restrictions as possible through good disease management. To manage their disease, persons affected by T2DM needed knowledge from their doctors or specialists or from self-education.

*“As I just said, you get yourself some material. And get some reading material from the doctors, which they provided me. And I read everything myself, and then tried to do as it said.” (Participant 4, male, 73 years old)*

*“So, my general practitioner has explained to me, what influence for example my diet or physical activity has on the diabetes and the interactions, what happens in the body with the blood glucose and how can it be regulated with the medicines.” (Participant 16, male, 47 years old)*

Our participants described very different barriers to learning. A great deal of individual initiative seemed to be required, and patients did not necessarily perceive their health insurance companies as supportive. Another barrier to learning seemed to be scheduling times and dates for patient education and training. Working people, in particular, described problems arranging regular training sessions, which were frequently held in the afternoon. Training seemed to be time consuming and related to the amount of time required for care, such as regular check-ups with T2DM and other specialists, meal preparation and physical activity.

*“Not at all, that's all personal interest and commitment.” (Participant 18, female, 60 years old)*

*“The course with the specialist doctor wasn't bad, either, but because of work I could only go every 14 days. So I missed half of it.” (Participant 16, male, 47 years old)*

Participants seemed to perceive an insufficient number of blood glucose measuring strips as a significant barrier to learning. Most of our participants possessed a glucometer supplied by their doctor. Regular self-monitoring for insulin treatment was standard practice, but some participants reported monitoring more often than doctors recommended or considered necessary. This additional measurement was often not compatible with the number of measuring strips supplied by doctors for self-monitoring, and the cost of measuring strips could influence these patients’ approaches to treatment. The problem seemed to be even more serious for patients who had received a glucometer but whose oral antidiabetic therapy did not provide self-monitoring of blood glucose. The effects of physical activity or certain foods seemed to be difficult to interpret for these patients, and they reported feeling not well informed about their condition; indeed, some seemed to suffer from this loss of control. These persons felt hindered in learning because they could not determine and interpret their blood glucose levels as fully as they wished.

*“No, no, that's because of the not very cheap measuring strips in there, that is. So, I do it at certain times when I think again, how was it today or something, but just not regularly.” (Participant 3, male, 49 years old)*

*“You get a month, no, in a quarter by 50 pieces. And if you buy strips, then each pack is about 25 euros, yes? This is natural, of course, that is much money, of course. So, I always try to get measuring strips somewhere else.” (Participant 7, male, 56 years old)*

### Implementation process: “Follow recommendations very strictly” – “Free design as a competent patient”

In the narratives of our participants, we identified two courses of action. One course seemed to be following the daily recommendations to the letter. The patients who did this seemed to have the highest regard for the authority of their physicians. These participants explained in detail how they have changed their lifestyle and how they have implemented the therapeutic requirements even though the result was a “life with prohibitions”, in which they saw more limitations than possibilities.

*“And here I eat cucumber, tomato, what I've grown in the garden myself, or lettuce, and actually it agrees with me, I'm managing well.” (Participant 1, female, 76 years old)*

*“And I have to discipline myself a bit, too, although it's hard, I admit.” (Participant 9, female, 80 years old)*

The other course of action seemed to be more flexible. These participants could be described as persons who did not want their T2DM to take over their lives, so they set their own rules and goals and tried to compensate for doing something that was not recommended. Even in training sessions, they thought about the means that they would and would not implement.

*“No, no, no, no, no, that makes even less sense, if you can't enjoy life anymore.” (Participant 12, male, 71 years old)*

*“I eat too, I really enjoy a piece of cake, and I used to [laughs] always have one on hand. And then you could compensate for it, for instance, by eating less in the evening or injecting more. But I did, what I could and I've always had everything under control.” (Participant 11, male, 64 years old)*

Both groups reported the same perception of check-ups. This time-consuming commitment seemed to be positive for patients because these check-ups could give them the certainty that nothing was wrong, and this assurance could be reassuring for patients who learn from fear and possibly relieve their anxiety. However, patients who interpret their actions more freely reported benefits from this confirmation as well.

### “Confidence in the action”

The action strategies “Follow recommendations very strictly” versus “Free design as a competent patient” seemed to be influenced either by patients’ confidence in themselves or the effectiveness of the interventions. On the one hand, some patients seemed to have a stronger external locus of control and prefer to blame genetics or external circumstances for their T2DM. These participants claimed that they were not always successful at disease management despite their efforts and that the measures that they learned did not necessarily work.

*“I inherited it too, the whole thing.” (Participant 17, male, 56 years old)*

*“And you see how the sugar goes mad sometimes.” (Participant 15, male, 50 years old)*

*“He [the doctor] also sees that he gets the diabetes or the sugar down now.” (Participant 8, female, 64 years old)*

On the other hand, some patients seemed to have a high internal locus of control and a high expectation of self-efficacy. These persons described taking some leeway in managing their T2DM to maintain their quality of life.

*“I am, and have, actually been able to deal with the diabetes well so far, without my/ It's not restricted me in any way, right? I've been able to join in all the parties and everything, and also to go abroad, it's all gone well, because everything was under control.” (Participant 11, male, 64 years old)*

*“I see that you may be able get around it here and there, but how you can manage it properly, that I just don't/ But I define it rather, and I take responsibility for it, so I say: yes, it's a fact.” (Participant 3, male, 49 years old)*

### “Acceptance and living with the disease”

The effects of the different learning processes and the actions or strategies that the participant developed could be described as “acceptance and living with the disease”. Ideally, persons affected by T2DM acquired sufficient self-management skills or could interpret these skills and were not severely restricted by their illness. These ideal conditions did not apply to all patients; information and knowledge alone did not induce changes in behaviour. In some cases, the learning process seemed to be delayed, especially for people who could not attend training sessions because of work and those whose management of their lifestyle and therapy had to accommodate their schedules. Patients also reported feeling unable to control the disease if they could not monitor it themselves, e.g., their form of treatment did not allow for self-monitoring so they have to buy their own blood glucose strips. Some of our participants could not afford them and seemed to feel guilty about not being able to monitor their blood glucose. The inability to monitor may have been associated with uncertainty and dissatisfaction.

### Educational inequalities in the results

In our study, we observed two courses of action: “follow recommendations very strictly” and “free design as a competent patient”. Furthermore, minor differences in educational status could be observed. Patients who were less educated tended to place more emphasis on being compliant, which goes hand in hand with a life with prohibitions and restrictions. In contrast, being perceived as competent patients who imparted more freedom to their lives and disease management appeared to be more important for people with higher education levels. The differences between the two courses concerned the implementation of new daily routines. Patients with less education described their new routines in detail, placing more emphasis on complying with the restrictions and prohibitions, and seemed to value the authority of their physicians. In contrast, well-educated people with T2DM seemed to want to be perceived by the research team as knowledgeable in daily life and in their interaction with the physicians. At the same time, the locus of control showed minute differences. Predominantly well-educated persons with a high internal locus of control and a high expectation of self-efficacy were found to take some leeway in managing their diabetes to maintain their quality of life.

## Discussion

The results presented here were from a study drawing attention to the perspectives of persons with T2DM in different care settings and considered the patient experience in Germany’s healthcare system. We gained insight into the different experiences of the disease, especially the perceived challenges in adapting to a diagnosis of T2DM.

In our study, adaptation of the previous lifestyle to a life with disease seemed to be central to the disease experience of persons with T2DM. Two possible ways of accepting the challenge of the adaptation processes dominated: 1. learning from fear and 2. learning such that life goes on well. These different motivations were associated with different emotions. Learning from fear was based on negative emotions, while learning was based on a positive, optimistic mood. Even though the relationship between learning and emotions does not seem as simple as positive emotions result in learning well and negative emotions result in learning badly [49, 50], some evidence indicated that positive affective processes underlie positive health behaviour change [51] and may beneficial in most cases [49]. Furthermore, two implementation processes were observed. One group described itself as very faithful to therapy and strictly adhered to the implementation of the expert recommendations in their daily living. These patients seemed to be more strongly tied to the authority of their physicians. The second group tried to deal more autonomously with T2DM and lead a responsible but well-organized life with the disease. Both groups seemed to be linked to different attribution styles; the first group tended to have a more external attribution style, whereas the second group had an internal style. The resulting lifestyles differed in that the group with external attribution styles had a life with rules and prohibitions. However, the persons with an internal attribution style had lives in which they felt less restricted by the disease and reported fewer perceived limitations in quality of life.

In 2008, Funnel and Anderson described the change in diabetes therapy from compliance to the self-management model [14]. In our study, the patient group with a lower level of education strongly emphasized their compliance, and the narratives from the patients indicated that some did not fully understand the opportunities and challenges of self-management. With patients following recommendations so strictly, living with diabetes meant a greater limitation for them than for patients who managed their disease more freely. Whether patients who value compliance did not accept the self-management model or whether it was not offered to them by the doctor remained unclear. Evidence has shown that people with low health literacy were perceived by healthcare professionals as uninvolved and less motivated, as well as having less understanding of self-management [12]. On the basis of our study, we could not determine whether self-reported compliance actually led to good disease management behaviour or whether there were discrepancies with self-perception. The literature showed that secondary diseases and poorer process parameters were more frequently found in socially disadvantaged groups with lower socioeconomic status [33, 35, 52, 53], so that an effective health behaviour could not be assumed in principle.

The inductive approach brought the study result strongly towards self-management. However, not all dimensions that were categorized as self-management were reported by patients, making it difficult to classify these results into the extensive literature on self-management [6, 14, 26, 54]. When reporting on their experiences with T2DM and its treatment, patients seemed to place great importance on several components of self-management as they built the core phenome of their viewpoints. These components were physical exercise, blood glucose self-monitoring, and diet. However, other aspects of self-management, such as weight monitoring, foot care, and keeping a diabetes diary, were not mentioned. The stronger emphasis on diet, blood glucose monitoring and physical activity can also be found in other studies [55, 56]. These themes may simply be more closely linked to diabetes treatment for patients than, for example, foot care, and these issues may also be frequently discussed with doctors, families and the public. These reasons may explain why diet, physical exercise, blood glucose self-monitoring were so much in the focus of our participants. Looking more closely at the other topics (weight monitoring, foot care, and keeping a diabetes diary) and reinforcing their importance in training and consulting may be necessary.

Professional associations recommend that all persons with T2DM participate in structured patient education [15–18]. However, not every person with T2DM actually takes part in patient education training [57–59]. In our study, almost all patients reported earlier participation in training. However, the findings were interpreted and implemented in different ways. The learning processes for managing the disease proceeded very differently [8, 60]. The learning needs suggested that diverse social groups cope with T2DM differently and experience diabetes in various ways. Assessing how patient education addresses the different types of learning and whether all patients benefit equally from the training might be interesting.

In addition, the delivery of glucometers to patients who were not treated with insulin should be critically considered. The patient may assume that blood glucose monitoring is an important part of diabetes self-management. Although this practice is not consistent with medical guidelines, patients may think that they are not being treated adequately if they are not given testing strips, which may have tainted their perception of the medical care that they have received. To this end, there have been changes in the German healthcare system since we carried out our study. Glucometers are no longer supplied free of charge, although whether and in what form this action supports patient is currently still open.

Even though the research team performed the study with great care, the study has some limitations. First, we recruited patients only from Central Germany, in particular Halle/Saale and the surrounding area. Patients in other regions might have other experiences if the supply situation is different. Another limitation is that we did not have participants who had migrated from other countries. The proportion of immigrants in our region is not as high as that found elsewhere, especially in what was formerly West Germany, and we did not encounter them in the recruitment phase. However, as cultural factors seem to be a separate theme in immigrants’ health service utilization [61], we did not specifically look for immigrants in sampling. The third limitation is that due to the first screening in the doctors’ offices or hospitals, no statements can be made about patients refusing to participate. Our project was designed to explore the patient’s perspective, which also included the importance of family behaviour, the wider social environment and caregivers for dealing with the disease from the patient’s perspective. Due to the project planning, these important reference groups could not be specifically asked what constitutes a further limitation and will be addressed in subsequent projects. Lastly, we performed all steps of the grounded theory thoroughly. However, the process led us to results that give insight into different experiences of the disease, especially perceived challenges in adapting to the diagnosis of T2DM, even if we had not addressed the question so concretely. Nonetheless, the results described were obtained in a rigorous manner.

## Conclusion

The persons affected by T2DM who participated in our study seemed to have accepted the need for education and utilized the training offered by the T2DM management programmes. Different types of learning were associated with disparities in education. However, working people should have better access to T2DM education, and the needs of patients with lower educational levels should be considered. Different forms of structured diabetes training, which also take into account the conditions of modern life, are urgently needed in Germany. Further studies should analyse and evaluate patient education and apply it to different types of learners. Specific recommendations on T2DM self-management for patients with less education are necessary. The identification of different learning styles could clarify the association between socioeconomic and healthcare disparities. Some patients seemed to feel incapable of controlling their T2DM if they did not have glucose measuring strips. This observation requires general practitioners, specialists for diabetes, clinicians, and policy makers to ask whether those uncertainties are warranted. Should glucometers be available to everyone being treated for T2DM? These topics are important for further discussion.

## Additional file

Additional file 1: Derivation of categories. (PDF 598 kb)

## Abbreviations

CI_95_%95: Confidence-Interval
IDF: International Diabetes Federation
T2DM: Diabetes Mellitus Type 2

## References

[1] Zimmet PZ, Alberti KGMM (2016). Epidemiology of diabetes-status of a pandemic and issues around metabolic surgery. Diabetes Care.

[2] Tamayo T, Brinks R, Hoyer A, Kuß O, Rathmann W (2016). The prevalence and incidence of diabetes in Germany: an analysis of statutory health insurance data on 65 million individuals from the years 2009 and 2010. Dtsch Arztebl Int.

[3] International Diabetes Federation. IDF Diabetes Atlas: Eighth edition. 2017:2017.

[4] Jacobs E, Tamayo T, Rathmann W, Epidemiologie d. Diabetes in Deutschland. In: Deutsche Diabetes Gesellschaft und diabetesDE – Deutsche Diabetes-Hilfe, editor. Deutscher Gesundheitsbericht Diabetes 2017: Die Bestandsaufnahme. Mainz: Kirchheim + Co GmbH. 2017:10–21.

[5] Röckl S, Brinks R, Baumert J, Paprott R, Du Y, Heidemann C (2017). All-cause mortality in adults with and without type 2 diabetes: findings from the national health monitoring in Germany. BMJ Open Diabetes Res Care.

[6] Weitgasser R, Clodi M, Cvach S, Grafinger P, Lechleitner M, Howorka K (2016). Diabetes education in adult diabetic patients. Wien Klin Wochenschr.

[7] Rise MB, Pellerud A, Rygg LO, Steinsbekk A (2013). Making and maintaining lifestyle changes after participating in group based type 2 diabetes self-management educations: a qualitative study. PLoS One.

[8] Newton P, Asimakopoulou K, Scambler S (2015). A Qualitative exploration of motivation to self-manage and styles of self-management amongst people living with type 2 diabetes. J Diabetes Res.

[9] Powers MA, Bardsley J, Cypress M, Duker P, Funnell MM, Hess Fischl A (2015). Diabetes self-management education and support in type 2 diabetes: a joint position statement of the American Diabetes Association, the American Association of Diabetes Educators, and the academy of nutrition and dietetics. Diabetes Care.

[10] Levesque C (2017). Therapeutic lifestyle changes for diabetes mellitus. Nurs Clin North Am.

[11] Barlow J, Wright C, Sheasby J, Turner A, Hainsworth J (2002). Self-management approaches for people with chronic conditions: a review. Patient Educ Couns.

[12] Fransen MP, Beune EJAJ, Baim-Lance AM, Bruessing RC, Essink-Bot M-L (2015). Diabetes self-management support for patients with low health literacy: perceptions of patients and providers. J Diabetes.

[13] Gomersall T, Madill A, Summers LKM (2011). A metasynthesis of the self-management of type 2 diabetes. Qual Health Res.

[14] Funnell MM, Anderson RM, Feinglos MN, Bethel MA (2008). Influencing self-management: from compliance to collaboration. Type 2 diabetes mellitus: an evidence-based approach to practical management.

[15] National Institute for Health and Care Excellence. Type 2 diabetes in adults: management; 2015 [cited 2018 Nov 26]. Available from: URL: https://www.nice.org.uk/guidance/ng28. 26741015

[16] International Diabetes Federation. Recommendations For Managing Type 2 Diabetes In Primary Care. Brussels; 2017 [cited 2018 Nov 26]. Available from: URL: https://www.idf.org/e-library/guidelines/128-idf-clinical-practice-recommendations-for-managing-type-2-diabetes-in-primary-care.html.

[17] Bundesärztekammer (BÄK), Kassenärztliche Bundesvereinigung (KBV), Arbeitsgemeinschaft der Wissenschaftlichen Medizinischen Fachgesellschaften (AWMF). Nationale VersorgungsLeitlinie Therapie des Typ-2-Diabetes – Langfassung; 2013 [cited 2018 Aug 26]. Available from: URL: http://www.deutsche-diabetes-gesellschaft.de/fileadmin/Redakteur/Leitlinien/Evidenzbasierte_Leitlinien/dm-therapie-1aufl-vers4-lang.pdf.

[18] Powers MA, Bardsley J, Cypress M, Duker P, Funnell MM, Fischl AH (2017). Diabetes self-management education and support in type 2 diabetes. Diabetes Educ.

[19] American Diabetes Association (2015). Standards of medical Care in Diabetes—2015 abridged for primary care providers. Clin Diabetes.

[20] Deakin T, McShane CE, Cade JE, Williams RDRR (2005). Group based training for self-management strategies in people with type 2 diabetes mellitus. Cochrane Database Syst Rev.

[21] Duke SAS, Colagiuri S, Colagiuri R (2009). Individual patient education for people with type 2 diabetes mellitus. Cochrane Database Syst Rev.

[22] Ellis SE, Speroff T, Dittus RS, Brown A, Pichert JW, Elasy TA (2004). Diabetes patient education: a meta-analysis and meta-regression. Patient Educ Couns.

[23] Hermanns N, Kulzer B, Maier B, Mahr M, Haak T (2012). The effect of an education programme (MEDIAS 2 ICT) involving intensive insulin treatment for people with type 2 diabetes. Patient Educ Couns.

[24] Minet L, Moller S, Vach W, Wagner L, Henriksen JE (2010). Mediating the effect of self-care management intervention in type 2 diabetes: a meta-analysis of 47 randomised controlled trials. Patient Educ Couns.

[25] Pillay J, Armstrong MJ, Butalia S, Donovan LE, Sigal RJ, Vandermeer B (2015). Behavioral programs for type 2 diabetes mellitus: a systematic review and network meta-analysis. Ann Intern Med.

[26] Chrvala CA, Sherr D, Lipman RD (2016). Diabetes self-management education for adults with type 2 diabetes mellitus: a systematic review of the effect on glycemic control. Patient Educ Couns.

[27] Odgers-Jewell K, Ball LE, Kelly JT, Isenring EA, Reidlinger DP, Thomas R (2017). Effectiveness of group-based self-management education for individuals with type 2 diabetes: a systematic review with meta-analyses and meta-regression. Diabet Med.

[28] Vas A, Devi ES, Vidyasagar S, Acharya R, Rau NR, George A, et al. Effectiveness of self-management programmes in diabetes management: a systematic review. Int J Nurs Pract. 2017;23(5). 10.1111/ijn.12571 28758701

[29] Onwudiwe NC, Mullins CD, Winston RA, Shaya FT, Pradel FG, Laird A (2011). Barriers to self-management of diabetes: a qualitative study among low-income minority diabetics. Ethn Dis.

[30] Vissenberg C, Stronks K, Nijpels G, Uitewaal PJM, Middelkoop BJC, Kohinor MJE (2016). Impact of a social network-based intervention promoting diabetes self-management in socioeconomically deprived patients: a qualitative evaluation of the intervention strategies. BMJ Open.

[31] Horigan G, Davies M, Findlay-White F, Chaney D, Coates V (2017). Reasons why patients referred to diabetes education programmes choose not to attend: a systematic review. Diabet Med.

[32] Mielck A, Reitmeir P, Rathmann W (2006). Knowledge about diabetes and participation in diabetes training courses: the need for improving health care for diabetes patients with low SES. Exp Clin Endocrinol Diabetes.

[33] Grintsova O, Maier W, Mielck A (2014). Inequalities in health care among patients with type 2 diabetes by individual socio-economic status (SES) and regional deprivation: a systematic literature review. Int J Equity Health.

[34] Ricci-Cabello I, Ruiz-Pérez I, Olry de Labry-Lima A, Márquez-Calderón S (2010). Do social inequalities exist in terms of the prevention, diagnosis, treatment, control and monitoring of diabetes? A systematic review. Health Soc Care Community.

[35] Forssas E, Manderbacka K, Arffman M, Keskimaki I (2012). Socio-economic predictors of mortality among diabetic people. Eur J Pub Health.

[36] Geyer S (2016). Social inequalities in the occurrence of chronic diseases. Bundesgesundheitsbl Gesundheitsforsch Gesundheitsschutz.

[37] Gerlach S, Kulzer B. Soziale Ungleichheit und Diabetes. In: Deutsche Diabetes Gesellschaft und diabetesDE – Deutsche Diabetes-Hilfe, editor. Deutscher Gesundheitsbericht Diabetes 2017: Die Bestandsaufnahme. Mainz: Kirchheim + Co GmbH. 2017:216–25.

[38] Glaser BG, Strauss AL, Paul AT. Grounded theory: Strategien qualitativer Forschung. 3., unveränd. Aufl. Bern: Huber; 2010. (Programmbereich Gesundheit)

[39] Rathmann W, Scheidt-Nave C, Roden M, Herder C (2013). Type 2 diabetes: prevalence and relevance of genetic and acquired factors for its prediction. Dtsch Arztebl Int.

[40] Przyborski A, Wohlrab-Sahr M. Qualitative Sozialforschung: Ein Arbeitsbuch. 4., erw. Aufl. München: Oldenbourg; 2014.

[41] Maier W, Holle R, Hunger M, Peters A, Meisinger C, Greiser KH (2013). The impact of regional deprivation and individual socio-economic status on the prevalence of type 2 diabetes in Germany. A pooled analysis of five population-based studies. Diabet Med.

[42] Müller G, Kluttig A, Greiser KH, Moebus S, Slomiany U, Schipf S (2013). Regional and neighborhood disparities in the odds of type 2 diabetes: results from 5 population-based studies in Germany (DIAB-CORE consortium). Am J Epidemiol.

[43] Mielck A (2005). Soziale Ungleichheit und Gesundheit: Einführung in die aktuelle Diskussion.

[44] Kivimaki M, Virtanen M, Kawachi I, Nyberg ST, Alfredsson L, Batty GD (2015). Long working hours, socioeconomic status, and the risk of incident type 2 diabetes: a meta-analysis of published and unpublished data from 222 120 individuals. Lancet Diabetes Endocrinol.

[45] Walker J, Colhoun H, Livingstone S, McCrimmon R, Petrie J, Sattar N (2018). Type 2 diabetes, socioeconomic status and life expectancy in Scotland (2012-2014): a population-based observational study. Diabetologia.

[46] Galea S, Tracy M (2007). Participation rates in epidemiologic studies. Ann Epidemiol.

[47] Arbeitsgruppe ‘Epidemiologische Methoden’ in der DAE der GMDS und der DGSMP. Messung und Quantifizierung soziographischer Merkmale in epidemiologischen Studien. Berlin; 1997.

[48] Strauss AL, Corbin JM (1990). Basics of qualitative research: grounded theory procedures and techniques. 1. Print.

[49] Pekrun R (1992). The impact of emotions on learning and achievement: towards a theory of cognitive/motivational mediators. Appl Psychol.

[50] Tyng CM, Amin HU, Saad MNM, Malik AS. The influences of emotion on learning and memory. Front Psychol. 2017;8. 10.3389/fpsyg.2017.01454 PMC5573739 28883804

[51] van Cappellen P, Rice EL, Catalino LI, Fredrickson BL (2018). Positive affective processes underlie positive health behaviour change. Psychol Health.

[52] Collier A, Ghosh S, Hair M, Waugh N (2015). Impact of socioeconomic status and gender on glycaemic control, cardiovascular risk factors and diabetes complications in type 1 and 2 diabetes: a population based analysis from a Scottish region. Diabetes Metab.

[53] Dalsgaard E-M, Skriver MV, Sandbaek A, Vestergaard M (2015). Socioeconomic position, type 2 diabetes and long-term risk of death. PLoS One.

[54] Gonzalez JS, Tanenbaum ML, Commissariat PV (2016). Psychosocial factors in medication adherence and diabetes self-management: implications for research and practice. Am Psychol.

[55] Graffigna G, Barello S, Libreri C, Bosio CA (2014). How to engage type-2 diabetic patients in their own health management: implications for clinical practice. BMC Public Health.

[56] Laranjo L, Neves AL, Costa A, Ribeiro RT, Couto L, Sa AB (2015). Facilitators, barriers and expectations in the self-management of type 2 diabetes--a qualitative study from Portugal. Eur J Gen Pract.

[57] Holt RIG (2017). Diabetes education, education and education. Diabet Med.

[58] Nam S, Chesla C, Stotts NA, Kroon L, Janson SL (2011). Barriers to diabetes management: patient and provider factors. Diabetes Res Clin Pract.

[59] Schafer I, Kuver C, Wiese B, Pawels M, van den Bussche H, Kaduszkiewicz H (2013). Identifying groups of nonparticipants in type 2 diabetes mellitus education. Am J Manag Care.

[60] Kneck A, Fagerberg I, Eriksson LE, Lundman B (2014). Living with diabetes - development of learning patterns over a 3-year period. Int J Qual Stud Health Well-being.

[61] Klein J, Hofreuter-Gätgens K, Ovd K, Janssen C, Swart E, Tv L (2014). Socioeconomic status and the utilization of health Services in Germany: a systematic review. Health care utilization in Germany: theory, methodology, and results.

