# Supplementary figures and images for: ‘Learning to shape life’ – a qualitative study on the challenges posed by a diagnosis of diabetes mellitus type 2
Source: Int J Equity Health. 2019 Jan 24;18:19. doi: 10.1186/s12939-019-0924-3 (PMC6346523; doi:10.1186/s12939-019-0924-3)

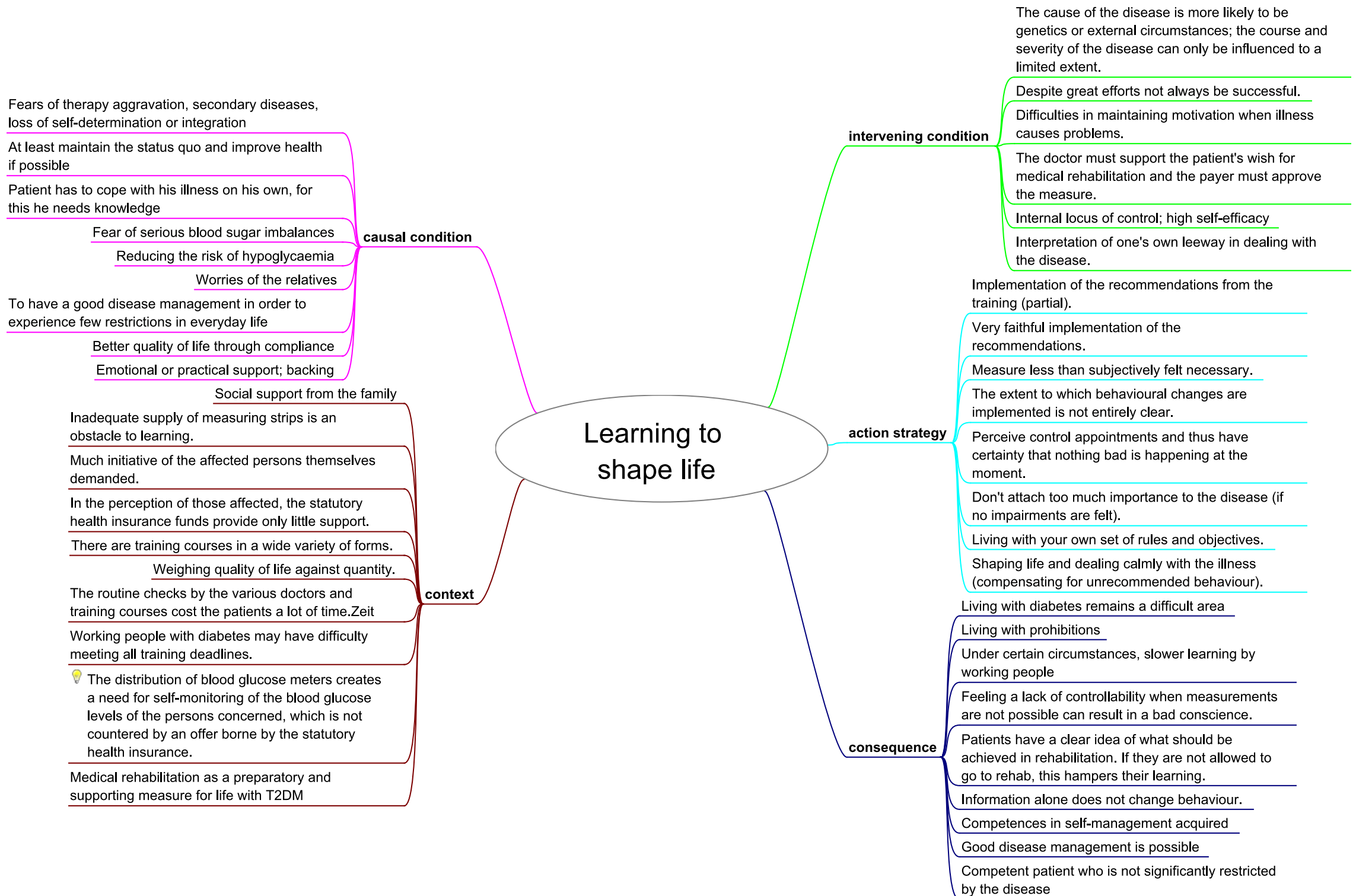

Supplement: Supplementary file 1 — Derivation of categories. (PDF 598 kb) [file 12939_2019_924_MOESM1_ESM.pdf]
